# Special Issue “Molecular Genetics and Plant Breeding 3.0 and 4.0”

**DOI:** 10.3390/ijms26052030

**Published:** 2025-02-26

**Authors:** Hai Du

**Affiliations:** 1 College of Agronomy and Biotechnology, Chongqing Engineering Research Center for Rapeseed, Southwest University, Chongqing 400715, China; haidu81@126.com or dh20130904@swu.edu.cn; 2 Integrative Science Center of Germplasm Creation in Western China (Chongqing) Science City, College of Agronomy and Biotechnology, Southwest University, Chongqing 400715, China

Molecular genetics aims to understand the genetic principles and functions of genes at the molecular level, while plant breeding aims to apply this information to improve plant traits and develop new crop varieties with desired characteristics. The advent of high-throughput sequencing technologies [1,2,3], e.g., next-generation sequencing (NGS) and third-generation sequencing technologies, allows for the rapid, cost-effective, and large-scale sequencing of entire genomes [4,5,6,7,8] and transcriptomes [9,10,11,12] in plants. Accordingly, multi-omics analysis strategies and bioinformatics methods are being widely applied in molecular genetics research, providing important information for plant breeding [13,14,15,16,17]. In 2019, the first Special Issue of the *International Journal of Molecular Sciences (IJMS)* on “Molecular Genetics and Plant Breeding” was launched to call for original research and review articles that present innovative research in plant molecular genetics, multi-omics, and gene resources at all levels, and 36 and 32 papers were published in the first two editions [18,19]. In the present third and fourth editions of the Special Issue, nine and twelve papers were printed, respectively. A fifth edition is currently in progress with a deadline on 27 August 2025 for manuscript submission.

DNA sequencing is foundational to numerous applications, such as genetic research, evolutionary studies, and crop improvement in plant breeding [14,15,16,17]. Seven papers published in this Special Issue focus on exploring the genetic basis, genetic variation, molecular markers, and functional genes in different plant germplasms and population materials. These studies employ a series of genome-wide analyses, including genome sequencing, genome-wide association studies (GWASs), quantitative trait loci (QTL) mapping, high-resolution molecular markers, etc. (Table 1, contributions 1–7). RNA sequencing (RNA-seq) is a fundamental tool in molecular genetics, functional genomics, and systems biology studies, providing a comprehensive and quantitative view of gene expression at the genome scale in various biological processes and with applications in diverse fields such as plant breeding. Four papers published in this Special Issue report novel results exploring the expression patterns, molecular mechanisms, and gene resources associated with different plant traits. These findings provide a foundation for molecular-assisted breeding (Table 1, contributions 8–11).

In the post-genomic era, applying bioinformatics approaches to rapidly and efficiently decode evolutionary or molecular information in plant genomes and to mine critical genes [20,21,22,23,24,25,26,27] represents an important issue. This Special Issue also covers the evolution, identification, and molecular analysis of gene resources in plant genomes. Three papers published in this Special Issue provide new insights into the genome-wide identification, evolution, and characterization of different gene families in plants (Table 1, contributions 12–14). Another three papers report the functional analyses of candidate genes involved in distinct bioprocesses in *Foxtail millet*, *Zea mays*, and *Sorghum bicolor,* respectively, using molecular and/or RNA-seq methods (Table 1, contributions 15–17).

This Special Issue also comprises four review papers (Table 1). The review by Zhang et al. (contribution 18) focuses on the synthesis and action mechanisms of non-coding RNAs and their roles in gene expression in plants. The review by Li et al. (contribution 19) summarizes the most recent advances in understanding the roles of functional endophytes in regulating plant secondary metabolism. It also discusses the potential applications and development of endophytes in agriculture, medicine, and industry. The review by Adhikari et al. (contribution 20) presents an overview of the MADS-box gene family members in plants and discusses their roles in plant organ development and trait-linked factors across diverse plant species. It concludes that most of the studied MADS-box genes are associated with flower- and fruit-related traits, and these family members play a significant role in angiosperm evolution. Sun et al. (contribution 21) review the recent advances in the complex modulation of potato dormancy and sprouting, highlighting the influence of environmental factors, carbohydrate metabolism, and intricate hormonal regulation on these processes.

In the past two decades, with the advent of high-throughput sequencing technologies and bioinformatics skills, the field of molecular genetics and plant breeding has become highly dynamic and is rapidly evolving. A total of 22 articles were published in the third and fourth editions of this Special Issue, including 17 original research articles, 4 comprehensive reviews, and this editorial. These papers highlight the recent advances in molecular genetics and plant breeding at the genome-wide level, focusing on diverse biological processes. They represent excellent contributions to the development of knowledge in these research fields and provide important theoretical insights and genetic resources for practical applications in plant breeding.

## Figures and Tables

**Table 1:** Compilation of the 21 contributions in the Special Issue.

Contributions	Species	Purpose	Approaches
1	*Prunus tenella*	Geographic variation and population genetic structure	Genetics, genomics, and transcriptomics
2	*Triticum aestivum*	Genomic regions associated with rust resistance	Genetics and genomics
3	*Zea mays*	New markers linked to fusarium resistance	Genetics and genomics
4	*Zea mays*	Genetic basis of plant architecture traits	Genetics, genomics, and transcriptomics
5	*Oryza sativa*	Genomic variation in tetraploid rice and its potential in breeding	Genetics, genomics, and bioinformatics
6	*Oryza sativa*	Genetic basis of lysine content	Genetics and metabolomics
7	*Hordeum vulgare*	Quantitative trait loci for *β*-Glucan content and grain traits	Genetics and physiology
8	*Salvia miltiorrhiza*	Molecular mechanism of pollen abortion	Transcriptomics, genetics, and molecular biology
9	*Malus domestica*	Genes involved in anthocyanin biosynthesis	Transcriptomics and molecular biology
10	*Diospyros kaki* Thunb.	Molecular mechanism of fruit size formation	Transcriptomics and molecular biology
11	*Pyrus pyrifolia*	Lignin biosynthesis and accumulation	Metabolomics, transcriptomics, and molecular biology
12	*Brassica napus*	Genome-wide characteristics of trehalose-6-phosphate synthase (TPS) gene family	Bioinformatics and genetics
13	*Vitis vinifera*	Genome-wide characteristics of the KCS gene family and its role in male sterility	Bioinformatics and transcriptomics
14	*Citrullus lanatus*	Genome-wide characteristics of the chitinase gene family and its expression profile	Bioinformatics and molecular biology
15	*Zea mays*	Transposition and repair mechanism of *Helitrons*	Transcriptomics, transformation, and molecular biology
16	*Sorghum bicolor*	Effects of epicuticular wax on anthracnose resistance	Transcriptomics, transformation, and molecular biology
17	*Foxtail millet*	Function of amino acid permease (AAP) transporter *SiAAP9* gene	Molecular biology and transformation
18	Plants	Roles and interactions of non-coding RNAs	Review
19	Plants	Functional endophytes in regulating secondary metabolism	Review
20	Plants	Functional characteristics of MADS-box genes	Review
21	*Solanum tuberosum*	Modulation of tuber dormancy and sprouting	Review

## Data Availability

For original datasets, please refer to the published articles within the Special Issue “Molecular Genetics and Plant Breeding 3.0” (https://www.mdpi.com/journal/ijms/special_issues/6W7Z125668) (accessed on 31 December 2023) and “Molecular Genetics and Plant Breeding 4.0” (https://www.mdpi.com/journal/ijms/special_issues/ZAM60GJ3Q7) (accessed on 15 February 2025).
